# Principles or Propositions of Medicine

**Published:** 1827-07

**Authors:** 


### PRINCIPLES, OR PROPOSITIONS OF MEDICINE.

By F. J. V. BROUSSAIS, M.D. &c. &c.

### PART. III.—THERAPEUTICS.

THERE is a strong disposition, both in and out of the profession, to regard therapeutics as the grand, if not the sole object of medical study. Thus the pupil will be seen yawning or sleeping while the lecturer is descanting on diagnosis, etiology, or pathology; but the moment the TREATMENT is mentioned, he pricks up his ears, and begins to take notes. It is in vain to tell the world at large that there is little chance of a cure, if the *nature* of the disease be not first ascertained—it is still more in vain to inform them that there are many diseases which it would be dangerous to cure, and still more which it is impossible to remove. In such cases, much harm may be done by blind and vague attempts with powerful therapeutical agents.

When, however, the proper basis is laid in anatomy, physiology, and pathology, PRACTICE will proceed on safe, if not always on successful grounds. If knowledge sometimes makes a man timid in the application of medicines, this is a much less dangerous error than temerity, grounded on ignorance. The former may not cure the patient, but the latter may kill him. With these few preliminary observations, we proceed to the third division of M. Broussais' aphorisms, embracing THERAPEUTICS.

PROP. 262. It is dangerous not to arrest inflammation in its early stage, since the efforts which nature makes to remove the malady are violent, and not seldom unsafe.

263. There are four kinds of means for arresting the progress of inflammations: viz. debilitants, revulsives, fixed tonics, stimulants, more or less diffusible.

264. The debilitants are, blood-letting, abstinence, diluent and acidulous drinks; but blood-letting is the most efficacious of all.

265. Venesection is the most proper mode of blood-letting, where sanguineous congestions form suddenly, under the influence of irritation, in the parenchymatous structure of organs:—In all other cases, *local bleeding*, as near as possible to the parts inflamed, (for instance, from the skin immediately over the principal seat of phlogosis) should be preferred, especially when the inflammation is recent.

266. There is no inconvenience in pushing blood-letting to the point of syncope, in a recent inflammation of a previously sound subject. Under opposite circumstances, we run a considerable risk by carrying sanguineous depletion to the above extent. The same may be said of abstinence. It is dangerous to make this too complete, or to prolong it too far, in unsound subjects, or where the inflammation has made progress. The bleeding from leech-bites is sometimes excessive in children, and in young people whose skin is florid and the action of the heart energetic. The bleeding, in such, should be arrested as soon as any symptoms of faintness occur.

267. Local bleedings are often hurtful in old-standing inflammations of the principal viscera, when there is no superabundance of blood in the system generally. It rarely happens that the local bleeding fails to increase the congestion in such cases. It is better to abstain from local bleeding under such circumstances, or else to draw the blood from a part at a distance from the principal point of irritation.

268 General or local blood-letting, in a person deficieut of blood generally, always produces much restlessness or malaise —augments the visceral congestions—and often gives rise to convulsions, and even fever.

269. When a recent inflammation, that had readily given way to sanguineous depletion, in a sound constitution, suddenly revives again, we may again and again have recourse to the same measures:—the convalescence will still be prompt; but if a chronic inflammation had pre-existed, this practice will be often dangerous. It will be also dangerous, if the inflammation had been general, or in two or more organs at one time. In such cases, we must stop depletion, if the pulse loses force, even if it maintains its frequency.

270. Slight inflammations of the encephalon readily yield to leeching of the epigastrium, especially if any gastric inflammation had preceded the encephalitis. But strong congestions of the brain require general bleeding from the jugulars, temporal arteries, or the arm; together with leeches to the upper parts of the neck—cold to the head; and hot pedi-luvia.

271. Cerebral congestions, with feeble pulse, require cold applications to the head, and rubefacients to the lower extremities, before having recourse to blood-letting.

272. Leeches placed on the inferior portion of the neck, between the insertions of the sterno-mastoid muscles, remove bronchial catarrh, and thus often prevent phthisis. This mode of leeching is very efficacious in the catarrh which accompanies measles, and which sometimes proves fatal, if not checked. The puriform nature of the expectoration ought to be no bar to this application of the leeches.

273. Leeches applied about the clavicles, and under the arm-pits, arrest the catarrhal inflammation which may have spread to the superior lobes of the lungs, and which generally terminates in phthisis, unless early removed. A dull sound in these parts indicates that the inflammation has reached the pulmonary parenchyma, and imperiously demands local bleeding.

274. Leeches applied to the epigastrium more effectually remove gastric inflammation, than when applied to the anus. But this last site of their application is more beneficial in colitis.

275. When colitis does not cede to anal leeching, and when a point of pain and tumefaction can be distinguished in the track of the colon, blood should be taken from this point, by leeching or cupping, which will effect a cure.

276. To remove incipient colitis by proper local bleeding, is to annihilate epidemic dysenteries.

277. Cynanche tonsillaris, cyn. pharyng. and cynanche trachealis are better removed by local bleeding than by emetics, which often exasperate the complaint, especially where there is plethora or gastric inflammation.

278. Symptoms of biliary and gastric irritation are sooner and more certainly removed by leeches to the epigastrium, or merely by abstinence, with water for drink, than by emetics.

279. Jaundice almost always depends on inflammation of the mucous membrane of the stomach and duodenum, or of the liver or its ducts, and is best removed by leeches applied between the epigastrium and hypochondrium, and by strict abstinence.

280. Articular inflammations cede readily to local bleeding, when they are strictly local themselves; but if they are complicated with gastric irritation, it is often necessary to apply leeches to the epigastrium, to accelerate their cure.

281. Eruptive fevers, being the signs of inflammation of internal organs precursory of that of the surface, local bleeding from the nearest vicinity of the internal irritation, renders the eruption more easy, and diminishes the danger.

282. The *secondary* fever of confluent small-pox, being the effect of the erysipelatous inflammation produced by the pustules, may be moderated, and sometimes prevented, 1mo, By the local bleeding practised for the *eruptive* fever; and 2ndo, By leeches applied to the neck, immediately previous to the inflammation of the face.

283. The fever called *adynamic* (or typhoid) which supervenes on confluent small-pox, being no other than gastro-enteritis, produced by the cutaneous inflammation, may be prevented, generally, by the means recommended for checking the excess of the cutaneous phlogosis.—*See Prop.* 232, *and* 233.

284-5. The intestinal worms which accompany entero-gastric inflammation, being only the effects or consequences of this phlogosis, require no specific means of cure beyond those necessary for the removal of their causes.

286. The *sequelæ* of measles are inflammations of the bronchia, of the lungs, or of the primæ viæ; and therefore require no other treatment than that of such phlogoses happening under other circumstances.

287. Emetics do not cure entero-gastric inflammations (always remembering that high degrees of *irritation* and inflammation are considered by M. Broussais as synonymous) except by revulsion, and by the critical evacuations which they provoke. Their effects, therefore, are uncertain in slight cases; and in severe cases they are dangerous, as they increase the phlogosis if they do not succeed in removing it. The same may be said of purgatives. The bitter purgatives increase the febrile heat, while the saline medicines of this class mask the phlegmasia, and render it chronic. Such are often the effects of calomel and neutral salts, which only give a temporary relief by the diarrhœa which they induce—the latter frequently ending in marasmus or dropsy.

288. Blisters often augment entero-gastric inflammation, by an extension of the phlogosis from the surface to the mucous membrane of the stomach and bowels. They are not, therefore, so serviceable in adynamic (low) fevers, as they are supposed to be.

289. Blisters very frequently exasperate inflammations of the lungs, if applied previous to sanguineous depletion, whether the complaint be acute or chronic. Applied after depletion, they are very efficacious.

290. The stomach is an organ which requires stimulation, in order to keep up, by its sympathies, the necessary degree of excitement in other organs and their functions:—But this stimulation should be adapted to the degree of susceptibility in the stomach, for this is the seat of an *internal sense* which regulates the animal economy.

291. When the sensibility and irritability of the stomach are above the natural medium, all stimulants are injurious, as precipitating the play of the various functions, even to the point of annihilating them. Such is the case in intense gastritis, in chorea, and in yellow fever, &c.

292. Excessive irritability of the stomach does not always manifest itself by *pain*, nor by *vomiting;* but rather by the violence of the general fever, by delirium, by stupor, and by convulsive movements:—These sympathetic symptoms should warn the practitioner against the employment of stimulants.

293. When the stomach is tormented by stimuli, it sometimes disembarrasses itself of the irritation, by throwing an increase of action (by means of its sympathies) on the exhalent and secretory vessels:—hence it is that gastro-enteritis, thus improperly treated, is not always mortal.

294. When the stomach is affected with a chronic inflammation, of a certain degree of intensity, and occupying the whole extent of its mucous membrane, stimulants are peculiarly detrimental, and the organ cannot disembarrass itself of the irritation they produce, except by exciting general fever, when a crisis is sometimes effected.

295. The stomach affected with chronic gastritis, to which is added, increased irritation by stimulants, is in great danger, if it is unable to free itself by revulsion, in consequence of the intensity of the phlogosis. Hence the mischief often produced by mineral and other medicinal waters, &c. &c. The irritation which is thus sometimes projected to the lungs, brain, and extremities, is converted into phthisis, mania, apoplexy, or gout.

296. If chronic gastritis be circumscribed to a part of the stomach, its exasperation is generally evinced by *pain* during digestion, (especially if stimulants be taken) by malaise, and even by some feverishness: but if, by proper diet and tranquillising remedies, these points of inflammation be soothed, the sound portions of stomach will crave for food, and even for stimuli, by which the organ will appear, for a time, to be comforted. Presently, however, the phlogosed points will be irritated anew, and the original train of symptoms will be recalled.

297. In partial gastritis, many years will pass in alternations of irritation and tranquillity, according to the various modes of treatment employed, till, at length, the coats of the stomach are changed in structure, either by scirrhus, mollescence, or ulceration—and then death is the inevitable result.

298. Partial irritations of the stomach, characterised by the march indicated in propositions 296, 7, will be cured by perseverance in the mildest and least irritating food—by avoiding all stimulant medicines—by mucilaginous diluents. This cure sometimes requires years for its completion; but it is the only permanent cure. It may succeed, even when some degree of disorganization has taken place. But it is necessary not to debilitate the stomach by too much bleeding, nor by such extreme abstinence as may risk the loss of all digestive power in the organ.

299. In chronic-gastritis, and entero gastritis, not complicated with colitis, a cure is sometimes effected by combating the constipation by means of calomel and the neutral salts:—But this is only in cases where the phlogosis is slight; for if it be severe, and especially if the organization of the stomach be compromised, the cure will only be palliative, as is the case where other stimulants are used.

300. Hæmorrhoidal irritation is frequently the effect of chronic gastritis, or entero-gastritis, and ought to be treated in the same manner as these complaints. The exasperation of the gastritis may suppress the hæmorrhoidal, as it suppresses the menstrual flux, and it is then a great error to use stimulants, with the view of reproducing these discharges. The safest way is to direct the treatment to the gastric affection; for, this being removed, the hæmorrhoids will either be cured at the same time, or the discharge will return, if it be necessary to the constitution.

301. When the stomach is not sufficiently stimulated by aliments, all the functions of the body languish; but, in no long time, the hunger develops, in this organ, an irritation which excites several functions in a manner unfavourable to the preservation of the individual. Such is the fury or mental exaltation of people who attempt to starve themselves. 302. Hunger, therefore, excites gastritis, and this phlogosis calls up its usual sympathies.

303. The sense of heat in epigastrio, the pains of the head and limbs, and the redness of the tongue produced by hunger, are dispelled by food, when they are only in a certain degree of intensity;—at a later period these phenomena are exasperated by food, and, therefore, only bland liquids should be first given, and the lightest nourishment should precede any stronger alimentation. Bleeding should not be employed for the phlogosis produced in this way.

304. When food passes not properly digested from the stomach into the intestines, it there excites colic and diarrhœa, which cede to wine and alcoholic stimulants. If these are given immediately on the appearance of the colic, the digestion is re-established, and diarrhœa will be prevented. These facts prove that assimilation (digestion) goes on in the intestinal canal.

305. Imperfect digestion of the aliments often takes place during the treatment of partial chronic gastritis, by the soothing method; (par la methode adoucissante;) but the sympathies called up by this indigestion should not be considered as inflammatory. The treatment indicated in proposition 298 should be steadily pursued.

306. It is only when light food can be digested, that we can pronounce chronic gastritis to be cured.

307. He who does not know how to manage irritability of the stomach, will not know how to treat any disease. A knowledge of irritation and inflammation of the stomach and intestines is the key to pathology.

308. When pulmonic inflammations have resisted the usual antiphlogistic measures and blisters, they may yet be controlled efficaciously by cauteries, by setons, and by moxas, applied, as nearly as possible, to the seat of the disease. But this will not always hold good, when the inflammation is seated in the mucous membrane of the digestive tube.

309. Incipient acute hepatitis should be treated by active local bleedings, which remove the gastro-enteritic inflammation with which the hepatitis is almost always accompanied.

310. Chronic hepatitis is sometimes palliated by emetics, by purgatives, by calomel, &c. but it is rarely cured by any other means than a steady perseverance in low regimen, assisted by counter-irritation and issues in the neighbourhood of the organ.

311. Jaundice, unaccompanied by pyrexia, is most commonly the effect of gastro-duodenitis; and is more effectually cured by the remedies of this phlegmasia, than by purgatives and the pretended solvents. This rule is still more absolute when the icterus is accompanied by febrile action in the system.

312. Peritonitis, in an early stage, is readily removed by leeches, applied to the abdominal parietes; but when the inflammation has continued several days, it is often beyond the power of art. General bleeding rarely cures this disease.

313. Puerperal peritonitis being generally an extension of inflammation from the uterus to the peritoneum, ought to be arrested in the beginning by numerous leeches to the hypogastrium. Emetics generally exasperate the complaint.

314. The warm bath does not cure peritonitis unless it causes a revulsion on the surface. If it fail to do this, it increases the malady. This rule, however, does not apply to emollient fomentations.

315. The warm-bath often exasperates the acute gastro-enterites; because stimulation of the surface is commonly propagated to the internal organs and tissues. Cold applications to the abdomen (the lungs being unaffected) are more beneficial than warm, in phlogosis of the mucous membrane of the stomach and bowels. These applications sometimes prevent repetitions of the bleedings.

316. When inflammation seizes, at the same time, on the mucous membranes of the lungs and of the primæ viæ, we may, after bleeding, apply cold to the abdomen, and warm cataplasms to the thorax. But if, under this treatment, the cough gets worse, we must discontinue the cold applications to the abdomen.

317. Typhus fever being gastro-enteritis, produced by the action of miasmata, often accompanied by other local phlegmasiæ, and especially of the brain or its membranes, may be arrested by the treatment proper for these inflammations, if taken at a very early period.

318. When the inflammation of typhus is not attacked in the earliest period, sanguineous depletion is often dangerous; because the poisonous miasma that originated the phlogosis enfeebles the vital powers and the living chemistry in such a manner that the loss of blood cannot be easily repaired.

319. The excessive exaltation of the vital phenomena (or in other words, the excessive excitement) is the most potent cause of their subsequent depression; and heat is the agent most capable of producing or increasing the primary excitement. Hence the fevers of hot countries, (where, besides, the miasmata are most poisonous,) are the most dangerous of all, and destroy the robust and vigorous more quickly than the weak. We are justified in concluding that cold applications in tropical fevers would be more efficacious than repeated bleeding; but this agent should be employed from the very commencement, immediately after bleeding—and internally as well as externally.

320. The slightest stimulant augments the intensity of fevers of hot countries, when exhibited at an early period. Emetics are then very dangerous—for example, in yellow fever.

321. As acute inflammations are much more rapid when they supervene on chronic, the most efficacious means of diminishing the ravages of yellow fever would be such as prevent chronic gastritis, (which is often the prodrome of acute inflammation in the same parts) and render the individual acclimaté.

322. Acclimation to a hot country is obtained by general blood-letting—by abstinence—and by quietude;—but we should avoid the abuse of vegetable aliment and refrigerant drinks, which produce indigestion, this last induces gastric irritation, the basis of gastric inflammation.

323. Free living is dangerous, in hot climates, for the newly arrived, since it induces a prolonged action of the stomach, which keeps up an increased flow of blood to that organ. The same observation applies to the abuse of alcoholic drinks. Excesses, therefore, in eating and drinking retard acclimation, and facilitate the operation of febrific miasmata on the body.

324. The ingestion of lightly spiced waters, with a slight addition of alcohol and vegetable acid should be used for the reparation of the fluids dissipated by excessive perspiration, in hot climates; but if the quantity of solid food be diminished to the proper standard, the thirst and perspiration will be much less considerable. 325. Concentrated stimulants are very injurious in tropical climates.

326. When prostration of strength has succeeded to high excitement in yellow fever, our principal resources will be found in acidulated drinks and glysters, and in cold applied to the exterior of the body, if the animal temperature be above par.

327. This proposition relates to the nourishment which is to be given in the last stages of typhoid fevers.

328. The nausea and vomiting which take place at the beginning of acute gastro-enteritis are not to be treated by emetics, but by leeches to the epigastrium, and hot applica tions to the lower extremities.

329. Constipation is favourable in acute entero-gastritis, (this term always meaning inflammation of the *mucous* membrane) as it indicates that the colon is not affected, and only requires one daily emollient lavement. If there be much heat of surface, this lavement should be cold.

330. Inflammation of the mucous membrane of the stomach, small, and large intestines, is cured by leeches in considerable numbers to the anus and neighbourhood:—but if there be much prostration of strength, and the vascular system of the patient appears in a state of anemia, we must content ourselves with rice-water or gruel, with gum and sugar, together with lavements of the same, containing some aqueous solution of opium.

331. When a prolonged discharge from leech bites, in the early stage of acute gastro-enteritis produces great debility, the practitioner should take care how he counteracts this by stimulants. The debility should be allowed to continue, as well as the discharge from the leech-bites, (unless the circulation become irregular) as the cure will be more certain, and the convalescence more rapid, after such a reduction. If syncope take place, of course, we must exhibit cautiously some light cordials, leaving them off the moment the circulation is restored.

332. If the bleeding from the leech-bites should continue, notwithstanding syncope and symptoms of asphyxia, we should stop the blood, especially in young children, who are more subject to die of hæmorrhage than grown people.

333. Local bleeding, abstinence, and diluents will always check incipient phlegmasia, if the inflammation be of no great extent in the organ affected. But if several organs be in a state of phlogosis at the same time, and to a considerable extent, as indicated by anxiety, restlessness, prostration, extreme frequency of pulse, we may then draw off all the blood of the body, and still leave the phlogoses in existence!—In such cases, we must not persist in sanguineous evacuations, merely because the pulse continues quick. We must economise the vital fluid and the animal strength, and restrict ourselves to the exhibition of aqueous diluents, adding gum or milk, if the tongue and teeth be not encrusted black.

334. An incipient meteorism (abdominal distention) in acute entero-gastrites, will be dispersed by a single application of leeches to the abdomen:—it is also cured by ice applied to the same part. If left unattended to, or if stimulants be given, it may change into peritonitis.

335. Subsultus tendinum and delirium, supervening on acute gastro-enterites, indicate an extension of the irritation to the encephalon, and if taken in the earliest period of their appearance, will often give way to leeches on the abdomen; but if these symptoms have continued some time, we must attack the encephalic irritation (now actually inflammation) by leeches to the temples, or what is still better, to the line of the jugular veins.

336. When the appetite returns vigorously in the course, or at the close of acute gastro-enterites, some indulgence should be allowed in broths and light soups, notwithstanding the frequency of the pulse, the heat, and the redness of the tongue;—otherwise the hunger will increase the gastritis, and bring back the stupor, black tongue, prostration, &c. But the indulgence in more substantial aliments would be injurious.

337. When, during convalescence, from acute gastro-enterites, pain in the head, bad taste in the mouth, nausea, malaise, and quickness of pulse take place, we may conclude that the convalescent has indulged too freely in eating. In such case, he should be kept almost entirely without victuals for a whole day, rather than exhibit emetics and purgatives. In the course of the following day, the convalescence will be re-established.

338. When, in the course of gastro-enteritis, there supervenes cynanche parotidea, we should moderate this symptom by the application of leeches, if the patient be not anemic, (bloodless from depletion) otherwise this external inflammation may renew the internal, or risk a congestion about the head.

339. When, in the course of gastro-enteritis, there supervenes a difficulty of making water, it is owing to an extension of the irritation to the bladder, and a prompt application of leeches to the hypogastrium will completely relieve this symptom, and prevent a host of accidents.

340. Epistaxis taking place in the course of acute gastro-enteritis is favourable, if the frequency of the pulse diminishes. If the hæmorrhage become excessive, we should restrain it by a blister to the nucha, or between the shoulders.

341. If hæmoptysis take place during acute gastro-enteritis, in spite of depletion, it requires a blister to the upper part of the sternum. Intestinal hæmorrhages require blisters to the abdomen, gummy drinks, or rice ptisans, acidulated with sulphuric acid.

342. Phthisis will be best prevented by early checking all inflammatory action in the respiratory apparatus.

343. We will cure HYPOCHONDRIASIS, and prevent scirrhous affections of the stomach, as well as pulmonary phthisis, by those means which remove chronic gastro-enteritis. Exercise of the body and amusement of the mind stand in the first rank of therapeutic agents in the cure of hypochondriasis.

344. Engorgements of the liver will be prevented or cured by the same means which cure chronic inflammation of the gastro-intestinal mucous membrane.

345. Chronic gastro-enteritis is to be cured by the lightest species of food, and by small quantities, at short intervals, of mild aqueous demulcent fluids.

346. We must not treat by *repeated* bleedings, and by *extreme* abstinence, chronic gastro-enteritis, except in otherwise robust subjects; because such treatment would cause an extent of debility that would require years for its removal, during which period, the irritability would be great, and the patient liable to frequent relapses. But mild aliment and demulcents taken during digestion (in the intervals of meals) will almost always cure the disease, if the viscera be not organically affected. The patient should be warned that the cure will be tedious, but that this plan of cure is the only effectual one.

347. Riding is dangerous in chronic gastritis, (what Dr. Philip terms the second stage of indigestion) where there is considerable exaltation of the sensibility of the stomach.

348. The air of large cities is injurious to persons affected with chronic gastritis; while that of the country is highly advantageous, especially if combined with exercise. The country air and exercise accelerate the digestion of the food, the prolongation of such process having always a tendency to keep up irritability in the stomach and intestines.

349. Vomits, purgatives, and tonics only procure temporary relief in chronic gastritis and gastro-enteritis, and render the radical cure more difficult.

350. The various mineral waters, whatever may be their temperature or their composition, seldom *radically* cure the chronic gastro-enterites; (indigestion and hepatitis) but often exasperate them. These temporary patchings up generally leave the patient quite incurable in the sequel.

351. Engorgements of the liver, spleen, and mesentery being almost always the effects of chronic gastro-enteritis, cannot be properly cured but by the means which cure this last disease.

352. The medicines and mineral waters which cause an evacuation of bile, of mucus, and of urine, or which excite perspiration, hæmorrhage, or cutaneous inflammation, diminish, temporarily, by a kind of revulsion, the engorgements of the liver and spleen, the irritation of the primæ viæ not being considerable; but it rarely happens that the above means operate a lasting cure. This can only be effected by a long perseverance in that abstemious system of diet and regimen, which suits the chronic inflammation or irritation of the digestive organs.

353. Chronic catarrh, with difficult expectoration of mucus from the bronchial membrane is *palliated* by the expectorants of authors; but it can only be *cured* by antiphlogistics, mild air, and by revulsion—(including counter irritation).

354. If we hope to prevent those scirrhous affections of the cervix uteri, which come on about the critical period (as it is called) in women who have been affected with painful menstruation, we should allay the irritability of the uterus long before the said critical period arrives.

355. The abuse of venereal pleasures being a frequent cause of cancer of the uterus, we should endeavour to reduce those chronic phlegmasiæ of the cervix uteri which lead to this deplorable malady.

356. It does not always require a long period for the formation of urinary calculi, renal or vesical:—but their formation might often be prevented by timely application of leeches to the region of the kidneys, and by the use of emollient drink, as soon as the nephritic symptoms make their first appearance.

357. Powerful diuretics, including the alkalies, turpentine, &c. often indeed expel those small calculi which have already formed in the urinary organs; but they too often exasperate that latent phlegmasia which produced them.

358. Recent catarrhus vesicæ yields readily to local blood-letting, to diluent drinks, to abstinence, and to the re-establishment of any cutaneous irritation which may have previously disappeared; but if it (catarrhus vesicæ) have become chronic, it is often incurable; and diuretics are then only palliative. Those means which produce most benefit, under such circumstances, are almost invariably of an antiphlogistic character.

359. Insanity cannot exist without some degree of irritation in the brain, accompanied by, and not rarely dependent on, chronic inflammation or irritation in the primæ viæ. The treatment therefore should be, local blood-letting, antiphlogistics, and revulsion. In abandoning these cases to nature, we expose the insane to epilepsy, paralysis, and apoplexy, which are the results of inflammatory disorganizations in the brain or its membranes. We expose our patients also to organic affections of the abdominal viscera, the inevitable finale of long-continued and neglected inflammation there.

360. Pulmonary phthisis, peritonitis, rheumatism, and gout, are only accidental diseases in mania :—not so phlegmasia of the mucous membranes of the abdomen, and engorgements of the abdominal viscera.

361. The principal distinctions of mania are not to be drawn from the kind of mental hallucination (nature de delire) but from the degree of organic irritation of the encephalon and digestive organs. The more inflammatory the graver the malady. The physical treatment must be based on the degree of physical lesion—the moral treatment on the nature of the hallucination.

362. Laryngeal and tracheal phthisis are always the effect of local inflammation not arrested in its early stages; and they do not prove mortal except by the supervention of pneumonia or gastro-enteritis. This misfortune, therefore, may be prevented by checking early the laryngeal or tracheal inflammation. If this be too far advanced, life may be procrastinated by preventing, as long as possible, the occurrence of pneumonia or gastritis.

363. Hypertrophia of the heart (not congenital) being often the effect of a latent phlegmasia of this organ, may be prevented by local and general bleeding, by digitalis, and revulsives.

364. Digitalis will not lower the pulse, unless the stomach be unaffected with phlogosis; and unless the principal viscera be also exempt from inflammation. Under opposite circumstances, this medicine accelerates the velocity of the pulse, and increases rather than checks the progress of inflammation.

365. Digitalis lessens the force of the locomotive muscles, and may therefore be advantageously employed in convulsions, provided no inflammation exists in any of the viscera. But in no case is it prudent to exhibit very large doses of this medicine, or for a long period of time.

366. Spontaneous hæmorrhages should be treated as inflammations, viz. by bleeding, general and local, by refrigerants, and especially by revulsion, whatever may be the degree of strength in the patient. Revulsion is the best resource where debility is considerable. 367. Spontaneous hæmorrhage is often kept up by a local inflammation, near or at a distance from the place of hæmorrhagy. 368. These hæmorrhages often coincide with hypertrophy of the heart. Digitalis may then be useful, provided the stomach be in a fit state to receive it.

369. Spontaneous hæmorrhage very often succeeds to inflammation, or takes on the character of phlogosis in the same place. We should therefore treat hæmoptysis as inflammation, by means of antiphlogistics and revulsives, without being restrained by the fear that tubercles pre-existed.

370. The mineral waters irritate and excite the heart and circulating system—augment the hæmorrhagic disposition, and even occasion hæmorrhages in those who are not predisposed to them, often determining aneurism, paralysis, or apoplexy.

371. Spasms and convulsions of every kind being the effects of a local irritation, fixed or ambulatory, cede to the treatment proper for such irritation—that is, to antiphlogistics, and sometimes to revulsion, where the irritated tissue is not disorganized.

372. Antispasmodics* will not cure convulsive affections, unless the stomach be capable of supporting them without being super-excited—and unless the local irritation, which is the cause, be under the degree of inflammation. On this account, the antispasmodics are often detrimental in hypochondriasis and hysteria.

373. Antispasmodics may suspend the phenomena of the nervous derangement, notwithstanding the inflammation of tissue, on which these phenomena depend; but the malady will be exasperated, and the cure will not be effected except through the medium of antiphlogistics and revulsion. Exercise of the voluntary muscles is the best mean of dissipating the disposition to convulsive movement and spasm in the system. It removes the visceral irritability and irritation by consuming them in another way and on another set of organs. It increases nutrition, and all the secretions.

374. Temperance is a condition, without which it is impossible to cure radically convulsive and spasmodic disorders.

375-6. Relate to the treatment of scurvy.

377. There are five modes of treating intermittent and remittent inflammations, now in use:—1mo, By antiphlogistics during the period of excitement—2ndo, By stimulants and tonics during the apyrexia—3tio, By stimulants during the febrile heat—4to, By stimulants administered at the very commencement of the cold stage—5to, By antiphlogistics during the apyrexia.

378. Intermittent inflammations cede to blood-letting and cold employed during the height of the hot stage, when the patient is robust and plethoric, and the disease recent;—in such cases, the leeches should be applied as nearly as possible to the principal seat of irritation.

---

* By the term antispasmodics, M. Broussais means only those which are of a stimulant nature; and not the *demulcents*, which he avers to be the best and safest antispasmodics.

379. Intermittent inflammations (fevers) yield, without danger, to bark and other tonics administered during the apyrexia, when there is no constitutional plethora, and when the principal viscera, especially those of digestion, present no trace of inflammation after the hot stage is over—that is, when the fever is clearly intermittent.

380. Intermittent inflammations are rarely cured by stimulants given during the stage of excitement. This plan is apt to change the intermittent into continued or remittent inflammation, or fever.

381. Intermittent inflammations (fevers) are rarely cured by stimulants given at the accession of the cold stage, because the excitement which they cause, augments the intensity of the hot stage. This plan rarely succeeds except after the employment of antiphlogistics, and in robust subjects, where the apyrexia is complete.

382. Inflammations with periodical exasperations, are cured by antiphlogistics, when some degree of inflammation (or irritation) continues in the viscera after the sweating stage; especially if this last be sufficient to keep up some pyrexia in the intervals—in short, when the fever is of a remittent character.

383. The surest plan of curing inflammations with periodical exasperations (remittent fevers) is to treat them at first by antiphlogistics, during the hot stage, and to continue this antiphlogistic treatment after the paroxysm, till the apyrèxia is complete—then to give the bark or other tonics during the whole period of the intermission. Diffusible stimuli, at the very commencement of the cold stage, may sometimes be allowed; but they are to be changed for cooling diluent drinks, when the hot stage sets in.

384. Bark and stimulants administered while there remains any inflammatory irritation in the digestive tube, may raise it into an acute and continued form—or they may stop the accessions, and establish a chronic and latent form of phlegmasia in those organs. It is in this way that bark produces *obstructions.*

385. Intermittent inflammations (fevers) abandoned to nature, undergo a spontaneous cure, when they are mild in degree, and when the cause ceases to be re-applied:—in opposite circumstances, they either change into acute continued inflammations, or they degenerate into a chronic state of continuity, accompanied finally, by visceral obstructions and dropsy.

386. Obstructions of the parenchymatous viscera (liver, spleen, lungs) sometimes supervene on intermittent inflammations, without any elevation of the gastro-enteritis, into a state of continued acuity. Such obstructions will be removed by the bark given during the apyrectic intervals.

387. When the bark stops an intermittent, and there supervene some degree of malaise, engorgement of the viscera, inappetence, and obscure febrile symptoms, we may conclude that the remedy was given too soon, and that chronic inflammation is going on in the mucous membrane of the digestive tube. This state is to be cured by antiphlogistics.

388. When, on the stoppage of an intermittent fever, there follows a bad condition of the digestive organs, the renewal of the fever (accidentally) by cold baths, or purgatives, is fortunate, if each crisis removes the irritation from the alimentary canal. If not, this renewal of the fever is a misfortune. 389. Relates to the proper mode of administering the bark.

390. Those intermittent fevers termed, from their fatality, *pernicious, &c.* can only be treated in the same manner as those to which this epithet is not applied—but we must be more prompt in using the remedies.

391. Dropsy sometimes shews itself at the very commencement of intermittent fevers; but it is more generally the result or consequence of their prolongation.

392. Dropsy produced by an obstacle to the circulation, yields to bleeding and gentle diuretics, provided the cause of the obstacle be not incurable. Digitalis is useful when this obstacle is hypertrophy of the heart.

393. Dropsy produced by the sympathetic influence of a chronic phlegmasia, is rarely curable, because such phlegmasia seldom occasions dropsy till the seat of the phlogosis is changed in its structure. The treatment must be directed almost solely to the chronic phlegmasia, and the diuretics should be such as do not irritate the digestive organs.

394. The dropsy which depends on an accidental defect in the urinary or perspiratory secretions or excretions will yield to the re-establishment of these, by the proper means. Diuretics and even purgatives will cure these; but we must take especial care to remove the accompanying vascular plethora, and not to exasperate the phlogosis which may co-exist.

395. Those dropsies which result from bad digestion and assimilation, disappear under the influence of tonics, good air, good aliments; but those which are caused by the abuse of mercury or other mineral substances, are often obstinate, on account of the gastro-enteritis which accompanies them, and sometimes causes them.

396. Dropsies resulting from hæmorrhages or other evacuations, are cured by tonics, good food, and active diuretics; but we should be very wary, in such cases, of too suddenly restoring the strength.

397. External scrophulous affections, unconnected with internal, may be removed by free application of leeches. The scrofulous diathesis, which is only an extension of irritation from tissue to tissue, will thus be checked. 398. Where this disposition to scrofula is not very inveterate, it may be removed by good air, and especially by exercise in the open air.

399. Stimulant ingesta will not cure the disposition to scrofula, except they excite the secretions, at the same time. If they do not excite the secretions and excretions, they aggravate the disease.

404. Chronic inflammation of the lungs (phthisis) is more rare than the chronic gastro-enteritis (tabes mesenterica) of infantile scrofulous subjects; because at this period of life, the lungs are less liable to irritation than the mucous membrane of the stomach and bowels. We should take care therefore not to add to this inflammation by food or medicine.

405. Syphilis is an irritation which, like scrofula, is propagated from the exterior to the interior. It may be stopped by vigorous antiphlogistics at the commencement. Even when become constitutional, syphilis may be eradicated by antiphlogistics and abstinence:—but the process being tedious and disagreeable, mercury and sudorifics are preferred.

406. Mercury cures syphilis by acting on the depuratory capillaries (secreting and excreting vessels) and thus causing a powerful revulsion. It should be seconded by abstemiousness.

407. Antisyphilitic medicines should be administered cautiously, as they are apt, if otherwise used, to occasion gastroenteric irritation, which, being reflected on the external syphilitic affections, aggravates instead of curing them.

408. When antisyphilitic medicines have produced enterogastric inflammation; and the syphilis remains, it will not yield till the entero-gastric disorder is removed by a long perseverance in abstemiousness and antiphlogistic remedies.

409. The gastric phlegmasiæ induced by the abuse of antisyphilitic medicines are readily transmitted to the lungs, and phthisis is the result, if abstinence and antiphlogistics be not promptly employed.

413. Syphilitic patients predisposed to gastric inflammation or irritation, should be treated on the antiphlogistic plan. If mercury be given internally, under such circumstances, the gastric irritation will be increased, and the syphilis itself often left uncured.

414. It is not safe to cure cutaneous eruptions attended with any degree of inflammation, by external stimulating applications. Antiphlogistics should first be employed.

417. The cure of intense phlegmasiæ, as of peritonitis puerperalis, acute rheumatism, pneumonia, &c. by tartar-emetic, calomel and opium, oil of turpentine, and drastic purgatives, is not effected by means of direct sedation; it results from the awakening of a great number of organic sympathies which open an extensive door to revulsion and critical evacuations. But if these stimulants fail in their purpose, they aggravate the original disease; and acute disorganization, or chronic inflammation is the result.

418. It is rare that the cure of severe morbid irritations by violent revulsive stimulants, is not followed by chronic irritation, and especially of the digestive organs. It is in this way that many cases of hypochondriasis are produced; for the stimulation of the stomach accumulates the sensibility of this organ, and gives more activity to the sympathies by which it is associated with the various other organs and parts of the body.

419 to 422. Relate to poisoning by various mineral, animal, and vegetable substances. They all act by producing irritation or inflammation first in the stomach and bowels, and reflected afterwards, by sympathy, to the encephalon, &c.

423. Debility is *most frequently* the product of irritation, and *sometimes* constitutes the whole disease.

424. Defective respiration is the most powerful cause of debility:—it produces necessarily abirritation (defective irritability,) but sometimes this is preceded by irritation.

425. In excessive spontaneous hæmorrhages, even without phlegmasia, the debility is always preceded by irritation; and finally becomes the principal malady. But in traumatic hæmorrhage, the debility is not preceded by irritation, and to this debility all our attention must be given.

426. The paralysis which succeeds to cerebro-spinal affections, is always the product of irritation. The same may be said of the paralysis which follows excessive discharges, not sanguineous.

428. Whatever may be the degree of debility attendant on irritations, it is to these last that we are solely to direct our attention, whenever they are of such a nature as to be aggravated by the ingestion of food or stimulant medicines. When they are under this degree, we may direct part of our attention to the debility, and part to the irritation which accompanies or causes it. Finally, when the irritation has ceased, and debility only remains, we are to be very careful in not too quickly removing this by too nourishing food, or too stimulating tonics.

429. Convulsions and pains, by whatever names they may be designated, leave behind them a debility, which *sometimes* furnishes the sole indication of treatment; but most commonly there is a degree of irritation left in the organ originally excited, which requires to be taken into consideration, when attending to the debility.

430. That debility which succeeds to venereal excesses, is always accompanied by irritation of one or of several organs.

431. Excessive external cold produces a debility which advances from the periphery to the centre, and may cause death. In these cases the debility constitutes the principal malady. But, if the external impression of cold be moderate, the vital powers excite, on the periphery, or in some of the organs, an irritation which becomes the principal malady, and which solely furnishes the indications of cure, when the external cold has ceased to act.

434. Those miasms which emanate from decomposed animal and vegetable matters, and from the bodies of congregated sick persons, are sometimes so deleterious as to occasion debility, and even death, without re-action; but whenever they produce pain and fever, there is established in the digestive organs (mucous membrane) and often (by sympathy) in other organs, an irritation, which furnishes the principal indication of treatment. This is what constitutes typhus—and is then the product of infection.

435. Every person affected with typhus may become a focus of infection for those who are well, and communicate to them the malady, if the patient be situated in a confined apartment, and the emanations proceeding from him become stagnant:—this is febrile contagion. But if the patient be placed in a well ventilated and clean ward, or chamber, this communication of the disease will be difficult. Are pestilential typhus and the eruptive diseases the only ones which can communicate the contagion, in spite of these precautions?

441. When violent gastro-enteritis is prolonged to a certain point, the debility is such as to furnish indications which we must attend to, lest the patient die of inanition; for an epoch arrives when digestion is possible, without exasperation of the symptoms, notwithstanding the persistance of the inflammation.

442. Those people, who have been a long time below that medium degree of embonpoint which comports with their constitution, require a long time to come up to that point again, with safety. They cannot support a certain vascular fulness without experiencing the effects of plethora, and risk of inflammation.

443. The sum of vital power diminishes in diseases of irritation, because the precipitation of the organic movements makes decomposition and elimination predominate over composition and absorption. We must except, however, certain *Bulimial gastrites*, where embonpoint and strength augment, in spite of irritation.

444. The indication of recruiting the strength by means of copious alimentation, is not to be drawn from the phenomena of emaciation or debility; but solely from the power of digestion and assimilation.

445. The indication of soliciting the stomach by means of tonics, is not to be founded on the debility or the emaciation of the patient, but rather on the paleness of the tongue, the languor, and the slowness of the digestion, after mild and unirritating aliments. Tonics are also indicated by certain pains in the stomach, by eructations, borborygmi, and colicky sensations that often accompany these slow digestions—all which phenomena disappear under a more stimulant alimentation, and proper tonics.

446. General debility, without any phlegmasia, requires only good food, with a moderate proportion of wine, if the stomach bears the latter without inconvenience. If the wine causes uneasiness, bitters should be employed.

447. Debility, accompanied by phlegmasia, situated elsewhere than in the digestive canal, requires light aliment which leaves but little feculence, the phlegmasia being acute. But this debility proscribes all stimulants. If the phlegmasia be chronic, the debility requires aliment of a substantial kind, but always of easy digestion. Tonics, in these cases, should be very light, and never long continued.

448. Debility with catarrh which exhausts by copious expectoration, unattended with fever, demands aliments substantial but easy of digestion, together with astringent tonics, in small doses, as the bark, the lichen islandicus, and the acetate of lead. Revulsives are here useful, but the suppuration should not be long kept up by counter-irritation.

449. The debility which accompanies acute gastritis requires the treatment necessary for such inflammation; but that which accompanies chronic gastritis demands farinaceous aliment, milk, and even the white meats, taking care to cool the stomach by demulcent fluids, when heated by the process of digestion.

450. The debility attendant on chronic colitis (dysentery) requires farinaceous food, deprived, as much as possible, of all feculence that may irritate the colon; and also a moderate proportion of red wine, that may tend to delay the aliment in the upper portion of the intestinal canal; for the irritation of the colon draws towards this portion of intestine the aliment before it is properly assimilated, and this produces the effects of a purgative.*

451-2. The debility produced by excessive hæmorrhages, and also that which succeeds to violent convulsions, (without gastritis,) require gellatinous, albuminous, and farinaceous aliment, with a small proportion of red wine, together with a cautious use of astringents and fixed tonics. But diffusible stimuli and high-seasoned food are injurious.

458. When, at the commencement of an acute disease, there exists extreme debility, accompanied by great mental depression, it is a sign that the inflammation occupies a large proportion of the respiratory or digestive organs, or both at the same time. At this period, if blood-letting, general or local, in place of relieving the debility, increases it, we should not repeat the depletion; since there is evidence that the organs of digestion and assimilation are not in a state to repair the waste of the system, much less any artificial depletion. Demulcent drinks internally—cold and counter-irritation externally, are the feeble resources of art on such melancholy occasions.

---

### COROLLARIES.

460. Empirical medicine, which consists in remembering certain symptoms which have been observed, and applying certain remedies that have been found useful, without any attempt at physiological reasoning in explication, is bad practice; because one single diseased or disordered organ produces a host of symptoms which combine with those dependent on

* For reasons which will be stated at the end of this article, we have avoided any comments on the therapeutical portion of M. Broussais' propositions: but we cannot help remarking here on the importance of proposition 450. Whoever has had the misfortune to be affected with chronic dysentery, will readily appreciate the justice of Broussais' statement; but few others will be sensible of its value. A small quantity of port wine, quietude, and gentle bitter tonics, will prevent the hurried passage of the imperfectly digested aliment along the upper bowels, and save the colon from unnecessary irritation. The writer of this article has found that small doses of the argentum nitratum will greatly reduce the undue sensibility of the small intestines, and conduce to the remora of the aliment there, till it is completely assimilated, and consequently till little is left for discharge through the colon.

other deranged organs, thus forming a variety of shades and modifications, in which it is impossible to accurately recognise the distinctive features or symptoms which are taken by the empiric for his model. We cannot practise safely, therefore, except by tracing the symptoms to the disorders of their respective organs.

461. But to practise medicine with success it is not sufficient merely to trace the symptoms to their organs; it is necessary also to determine in what respect these organs differ from a state of health—that is, we must ascertain the nature of the organic lesion.

462. The nature of the disease ought to furnish the practitioner with the indication of cure. The nature of the disease is ascertained, 1mo, from a knowledge of the agents which have exalted, diminished, or deranged the action or function of the organ primarily affected; 2ndo, from a knowledge of the influence which this organ exerts on other organs; 3tio, from a knowledge of those agents which have the power of restoring the equilibrium of function in organs, or controlling the intensity of disease.

463. The groups of symptoms which are given by nosologists as diseases, without referring them to the organs on which they depend—or without ascertaining the nature of the organic aberrations, are metaphysical abstractions which do not represent a morbid condition that is at all constant, invariable, or having a real model in nature:—they are artificial ENTITIES, and those who study medicine by this method, are ONTOLOGISTS.

464. To consider these factitious entities as morbific powers that act on the organs, producing this or that disorder, is to take effects for causes—is to construct ONTOLOGY.

465. To consider the succession of symptoms which one has observed, as the necessary and invariable march or progress of a disease, and thence to draw certain characters essential to the diagnosis and treatment, is to create a factitious ENTITY; since affections of organs differ in their symptoms according to the nature of the morbific agents. In short, it is placing ourselves in a condition where we cannot know the disease or its treatment till after it has run its course!

466. To apply remedies to an ENTITY, without appreciating their effects on the organs which receive them, and those which sympathise with these organs, is to cure or exasperate a disease, without knowing why or wherefore.

467. He who cures a disease, without having correctly appreciated the physiological modifications, by means of which he has effected this cure, has no certainty of being able to ascertain or cure the same disease when it next presents itself to his notice:—hence it is that neither the success nor the failure of the ontologist can render him a good practitioner, or enable him to teach others how to practise well.

FINIS.

The length of the therapeutical section of propositions, and our anxiety to finish it in this article, have forced us to avoid entering on any comments, in the form of notes, as we did in the physiological and pathological divisions. We were the less disposed to offer any remarks at present, in consequence of learning that the illustrious author was republishing the propositions with commentaries, through the medium of the Journal of physiological medicine. When his commentaries are complete, we shall dedicate an article to them in this Journal. In the mean time, we can now say that we have laid before the English profession a more comprehensive and exact view of M. Broussais's principles, than has ever before been done; and we are disposed to think that this exposé of a doctrine, which has now maintained its ground so long, and which is still extending its influence both on the continent and in this country, will be both useful and agreeable to our readers.

---

